# Legal Cases in Lunacy

**Published:** 1852-10-01

**Authors:** S. Vallis Bone

**Affiliations:** of Lincoln's Inn, Barrister-at-law


### LEGAL CASES IN LUNACY

AND IN CHANCERY, INVOLVING QUESTIONS OF INSANITY, ARGUED BEFORE THE LORD CHANCELLOR, THE LORDS JUSTICES OF THE COURT OF APPEAL, AND THE FULL COURT OF APPEAL IN CHANCERY.

*Reported exclusively for "The Journal of Psychological Medicine and Mental Pathology," by* S. Vallis Bone, Esq., *of Lincoln's Inn, Barrister-at-law.**

---

(Before the Lord Chancellor, November 4th, 1851.)

Price *v.* Berrington.

*Conveyance by a Lunatic supported notwithstanding the Lunacy.*

In 1809, a sale and conveyance of landed property was made for a particular sum of money. In 1836, a bill was filed on his behalf, alleging him to have been of weak mind since 1789, seeking to set aside the sale. In 1837, he was found lunatic by inquisition, and that he had been so from 1796, and on an issue directed by the Court the jury found that he was of unsound mind when he executed the conveyance. The purchaser having been in possession of the property twenty-seven years,

* These cases commence with the beginning of the legal year, 1851—1852, namely, Michaelmas Term, 1851.

and there being no evidence to satisfy the Court that he was aware of the insanity of the vendor, and the allegations of fraud failing, the Court refused to set aside the sale on the mere ground that the verdict of the jury carried back the lunacy to a date before that of the conveyance.

This case came before the Court in the form of an appeal from a decree of the late Vice-Chancellor, Sir James Wigram. The facts necessary to be stated are shortly these:—The Rev. Charles Price, by deeds dated the 5th, 6th, and 7th of February, 1809, conveyed an estate called Tyr-y-Caed-Cae, in the county of Glamorgan, to Mr. Moggridge, absolutely for 2000*l*. The whole purchase-money was not then paid; but in 1822 the balance was paid, and then, by deed dated the 19th of March, Mr. Charles Price, as "the eldest son and heir apparent of the Rev. Charles Price," confirmed the sale and released all his right and title to the estate. In 1836, a bill was filed by the Rev. Charles Price, "a person of weak mind," by Charles Price, his eldest son and heir-at-law and next friend, against the devisees under the executor of the will of Mr. Moggridge, who was dead, which, after alleging that the Rev. Mr. Price, the plaintiff, had, from imbecility of mind, been unable to manage his affairs ever since 1789; that Mr. Moggridge, knowing this incapacity, had fraudulently induced him to execute the conveyance; that the price was inadequate, and, moreover, that no more than 600*l*. was paid at the time, which was applied in discharge of a mortgage; prayed the re-conveyance of the estate and an account of the rents, the plaintiff offering to repay what had been paid for the purchase. The answers stated the payment of the whole consideration, part at the date of the transaction, and the remainder when the son executed the release; and the defendants said they believed the plaintiff had been subject to occasional temporary fits of insanity, but with lucid intervals, during which he was fully competent to manage his affairs, and that he was so at the date of the conveyance.

In the month of May, 1837, a commission of lunacy was issued, and on the 22nd of the same month a verdict was returned that the plaintiff was "a lunatic without lucid intervals, and was not sufficient for the government of himself, his messuages, lands, tenements, goods, and chattels, and that he had been in the said state of lunacy from the 1st day of June, 1796." After the return of the inquisition, Mr. William Price, the younger, was appointed committee of the person, and Mr. Charles Price, the eldest son, the committee of the estate of the lunatic.

After the finding of the lunacy, the proceedings in chancery were prosecuted by a supplemental bill filed in July, 1837, and on the 30th of June, 1840, the late Master of the Rolls (Lord Langdale) directed an issue to try whether "the plaintiff, William Price, was of sound mind at the time when he executed the deeds of the 5th, 6th, and 7th of February, 1809." Before the issue was tried, the lunatic died, in the month of January, 1841, and no further proceedings appear to have been taken until 1848, when Mr. Charles Price filed a bill of revivor and supplement; and by a decree, dated the 3rd of June, in the same year, the former order was directed to be executed, and the issue to be tried. The trial took place at Bristol Summer Assizes, 1848, when the jury found by their verdict that the Rev. Charles Price was not of sound mind when he executed the deeds in question. The cause came on upon further directions, and on the equity reserved, before Sir James Wigram, who made a decree, declaring in effect that the deeds were void, and directing certain accounts of the rents. From this decree both plaintiff and defendant appealed.

The Solicitor-General (Sir W. Page Wood), Mr. Bethell, Mr. Headlam, Mr. W. M. James, and Mr. E. F. Smith, were counsel for the several parties. On the point of the invalidity of the transaction on the ground of the insanity of the vendor, the following cases were relied on, and were also referred to at the close of the judgment:—Sergeson *v.* Sealey, 2, Atkyns' Reports, 412; Niell *v.* Morley, 9, Vesey Junior's Reports, 478; and Lewis *v.* Thomas, 3, Hare's Reports, 26.

The Lord Chancellor (Lord Truro).—This suit was instituted for a declaration of the Court that the conveyance of an estate in Wales was void on account of fraud and misrepresentation on the part of the purchaser, and on the ground that the vendor was a lunatic at the time he made the sale, and had been so from the year 1789. The plaintiff, who is the son of the vendor, placed his claim for relief on the ground of fraud under four different heads. The first was, that his father, the Rev. Mr. Price, had been insane for many years before the sale of the estate, in 1809, and that Mr. Moggridge (the purchaser) had full notice of that insanity; the second head was, that the consideration was invalid; the third was, that the value of the estate had been concealed from the vendor, inasmuch as there were valuable minerals underneath the soil, of which the purchaser had knowledge, and of which the vendor was ignorant at the time of the sale; and the fourth was, that violence and intimidation had been exercised on the vendor, at the instigation of Mr. Moggridge, to compel Mr. Price to execute the conveyance Now, if these charges are proved, there can be no reason for refusing the plaintiff that relief which he prays. The estate called Tyr-y-Caed-Cae is situate in Glamorganshire, and the conveyance was dated in 1809. The bill to set it aside was filed in 1836, by Mr. Price, the vendor's eldest son, claiming to be his representative, and charging fraud only; but a commission of lunacy being issued in 1837, and the Rev. Mr. Price being pronounced under the finding of the jury to have been a lunatic since 1796, the plaintiff then filed a supplemental bill, introducing the additional ground of insanity. The cause came on for hearing before the late Master of the Rolls in 1840, and he directed an issue on the question of insanity. That issue was tried, and the result was in favour of the plaintiff; but the Rev. Mr. Price having died while the proceedings were going on, it was not until 1848 that relief was prayed on the strength of that issue. The cause then came before Vice-Chancellor Wigram, who declared the deed of conveyance to be void. The plaintiff appeals on the ground that there ought to have been a decree on the original bill and supplemental bill without the formality of an issue; and the defendant appeals against the order of the Master of the Rolls for the issue, as well as against the order made on further directions by the Vice-Chancellor, and asks to have the bill dismissed with costs. It was argued, in the first place, on the part of the plaintiff, that the lunacy being established by the finding of the jury, that fact, without any other, entitled him to a declaration for setting aside the conveyance. I do not feel now that I am bound to give that point much consideration, or to express any very decided opinion on it. The Vice-Chancellor Wigram did not rest his decision solely on the fact of the lunacy; and at any rate the case comes before me under totally different circumstances from those under which it was heard on further directions in the court below. I have had in substance the whole of the merits brought under my attention by the appeals, and after a careful consideration of all the merits, and the facts in evidence, I am of opinion that the orders of the court below were wrong, and that the bill ought to have been dismissed with costs. Looking at the evidence of facts, it does not appear that Mr. Moggridge, the purchaser (whose daughter is now the defendant), had ever been in the company of the Rev. Mr. Price, or knew anything of his habits. There is no proof that he knew anything of the lunacy, and the other allegations are either of no importance, or not proved. The bill alleges that Mr. Moggridge bribed the wife of Price to intimidate and coerce her husband. That case has failed in proof;—the allegation of undervalue has failed;—the non-payment of the consideration has failed;—and it, moreover, appears that the affairs of the lunatic were at the time in such an embarrassed condition, that it was convenient for him to sell the property, there being a pressure for the payment of a mortgage debt secured upon the estate. There remains, then, the question of whether the mere fact of an insanity found twenty-seven years after the sale would invalidate that sale, there being no notice of the insanity brought home to the purchaser, and a failure of all proof of fraud; and that invalidity, too, is sought to be established against the children of Mr. Moggridge, who have been during the whole time in undisturbed possession and enjoyment of the estate. Such an interference with the rights of a *bonâ fide* purchaser would be contrary to principle, and manifestly unjust. The orders of the court below must be reversed, and the bill be dismissed with costs.

---

(Before the Lords Justices of Appeal. Dec. 3rd, 1851, and Jan. 15th, 1852.)

*In the matter of* Mr. James William Loveday.

*Costs of an Inquisition de lunatico inquirendo, which is superseded.*

The jurisdiction of the Court of Chancery is one of administering the property of a lunatic, and if that is gone there remains nothing in the hands of those who exercise the jurisdiction to administer. The statute 6 Geo. IV. c. 53, "An Act for limiting the time within which Inquisitions in Lunacy, Idiotcy, and *non compos mentis*, may be traversed; and for making other regulations in the proceedings pending a traverse," has made no difference, and the law stands as it was before in the case of a successful traverse of an inquisition. The Lord Chancellor or other persons acting in the jurisdiction of lunacy are not authorized either under the old jurisdiction or the 4th sec. of the statute to deal with the property of an alleged lunatic after the trial of a traverse finding such person not to be lunatic. The intention of the statute is to give the court power to act pending the traverse. For these reasons the court has no power to direct the payment of the costs of the inquisition out of the estate of the party found lunatic by inquisition, but declared not to be lunatic on a traverse, although the motives and conduct of the persons who sued out the commission are approved.

In this case, Mr. John Loveday and his wife, on the 21st of January, 1851, sued out a commission of lunacy against the wife's brother, Mr. James William Loveday, and the Commission was accordingly issued to inquire whether he was of unsound mind, and so as to be incapable of managing himself and his affairs, and if so from what time he had been so. The inquisition was held at the Shire Hall, at Gloucester, before Mr. Winslow, one of the Masters in Lunacy, on the 25th, 26th, 27th, and 28th of February, and 1st of March following, when the jury found that he was of unsound mind, and incompetent of managing himself and his affairs, and had been so from the 16th of November, 1850. On the 7th of April, Mr. James William Loveday presented a petition to the Lord Chancellor, praying leave to traverse the inquisition, and an order was made on the 28th of May, granting the prayer of the petition. The traverse came on for trial at the Gloucester assizes, on the 14th of August, 1851, and no evidence was given of the insanity of Mr. Loveday at any time, and it was admitted that he was then of sound mind, and by the consent of all parties the following verdict was returned—"The jury find that the defendant is now of sound mind, and sufficient for the government of himself, and his manors, messuages, lands, tenements, goods and chattels." After the finding of the original verdict, and on the 28th of

May, the same day when the petition of traverse was presented, the custody of Mr. Loveday and the care and management of his estate were granted to Mr. Charles Baker, for the time to come, and until further order, upon his giving such security as should be approved by the Attorney-General, such security to be perfected on or before the 10th of November following. This security was never perfected, and no further proceedings were taken under the order. After the traverse, Mr. Loveday presented a petition to the Lord Chancellor, which, after setting forth the abovementioned facts, and stating that Mr. Baker and Mr. John Loveday, or one of them, had then in his or their possession or control various title deeds and other property belonging to Mr. James William Loveday, taken possession of by Mr. John Loveday pending the proceedings, prayed that the commission and all proceedings taken under it might be superseded, and that Mr. Baker and Mr. John Loveday might deliver up to Mr. James William Loveday all deeds, documents, goods, and chattels of every description, in their or either of their possession, belonging to him. Before the traverse the Lord Chancellor had made an order directing the taxation of the costs of the commission and inquisition. As soon as the petition was opened it was stated by counsel, in answer to an inquiry from the court, that the supersedeas was not resisted.

The Solicitor-General (Sir Wm. Page Wood) and Mr. Amphlett, who appeared for Mr. John Loveday and Mr. Baker, were heard on the question of the costs of the commission. They asked that an addition might be made to the order for taxation, that the costs might be paid out of the estate of the traverser. They referred to the case of *ex parte* Ferne, 5 Vesey's Reports, page 832, to show the anxiety expressed by Lord Loughborough to give the costs to those who had properly conducted themselves in endeavouring to afford a supposed lunatic the protection of the Crown. They relied also on the 4th section of the statute 6 Geo. IV., chap. 53, as showing that the court had authority to interfere, and argued that as the Lord Chancellor had already made the order for taxation, which order went on to say that on the report thereon such order should be made as should appear just, the manifest intention was that all the reasonable costs should be paid. The property admitted to be in the hands of the respondents came to them in the course of the proceedings in lunacy, and it would be hard if they were obliged to deliver it up and be left to pay the expenses of the performance of a duty; the court would, therefore, at all events make the delivering up of that property only conditional and dependent on the payment of the costs by the traverser, even if it would not make an absolute order for payment of the costs, which would have the effect of a charge on the estate.*

Mr. Rolt and Mr. T. Terrell contended, that as there had been a successful traverse of the inquisition the Court had no authority whatever to deal with the property of the traverser, Mr. James William Loveday. The authority to give costs depended on the authority the Court had to administer the property of a lunatic, and that gentleman being found not to be a lunatic the power of the Court did not exist. They relied on the case of *ex parte* Ferne before cited, and remarked on the principle laid down by Lord Loughborough being admitted by Lord Eldon in the case of Sherwood *v.* Sanderson, 19 Vesey's Reports, page 280 They insisted, that the statute which had been cited made no difference in such a case as the present, the inquisition having been successfully traversed, the legislature carefully confining the authority to deal with the estate to the interval between the finding on the

* The effect of such an order, if it had been made against Mr. J. W. Loveday, would have been to create a charge on his estate; for, by the statute 12 and 13 Victoria, chap. 106, all orders of the Lord Chancellor, &c., in matters of lunacy, whereby any sum of money or any costs shall be payable to any person, are to have the effect of judgments.

inquisition and the traverse. If the property of the traverser could be dealt with, why might not his person be controlled? As to the suggestion, that the order for the delivery being made conditional, that would be useless, for Mr. James William Loveday would only have to bring an action for the recovery of the property, and the respondents would have no defence.

Their lordships reserved their judgment, Lord Cranworth expressing a desire to consider the point of costs.

January 15th, 1852.

Lord Justice Lord Cranworth now delivered the judgment of the Court, and, after detailing the facts of the case, proceeded thus:—The grant of the prayer of the petition, so far as it seeks a supersedeas of the commission, is a mere matter of course. A question then arises (which is the only question on which I wished for time) whether, as John Loveday, who sued out the commission, insisted, that it is a proper thing on the part of those who exercise jurisdiction in lunacy to give the costs of suing out that commission. That was disputed by James William Loveday, and that dispute gives rise to two questions: First, is there jurisdiction? and secondly, if there is, then is this a proper case in which to exercise it? With regard to the propriety of giving costs if there is jurisdiction to do so, that depends on the conduct of the parties suing out the commission; that is, how far they acted upon proper motives, and other questions of that description. Upon that, we had before us no information, and we took the opportunity of speaking to the Lord Chancellor upon the subject, from whom we heard that he was satisfied the parties had acted *bonâ fide*. Under the circumstances, therefore, if we have jurisdiction, we think the case a perfectly fit one in which to give the costs. Then arises the next and important question, whether we have jurisdiction. Now, there is no such jurisdiction independently of the recent statute 6 Geo. IV., c. 53. That was decided by Lord Loughborough in *ex parte* Ferne, in which case the form of the traverse was, that the party was a lunatic at the time of the marriage, and at the time of taking the inquisition. That was not the time in question. The time was that of the verdict being given, but the verdict was that at that time she was not a lunatic. The party not being then a lunatic, a supersedeas was of course. Then came the question of costs. The then Solicitor-General (Sir William Grant), and Mr. Fonblanque for the family of the Wraggs, pressed for costs, observing, that they had established a lunacy at the time. That appears to be a stronger case than the present, where it is left in doubt whether at the time of the inquisition the petitioner was a lunatic; the verdict found being simply that Mr. James William Loveday was not a lunatic at the time of the finding upon the traverse. In that case of *ex parte* Ferne, the Lord Chancellor (Lord Loughborough) wished to give the costs; but he said, "Where is the fund to pay the costs? Where the commission is superseded there can be no fund. There is a step to be taken, possession to be taken of the property. The traverse stops that. The lands and goods have never come into the hands of the Crown. The traverse is *de jure*. It is no favour. The parties apply by petition, stating that they are dissatisfied with the finding, and that stops the commission; there is no *amoveas manus* here. If I could act *cum imperio*, it is a very proper case; and the parties have entitled themselves to all the costs I can give them; but I have no jurisdiction."

That was the opinion of Lord Loughborough; and in a case of Sherwood *v.* Sanderson, the principle was fully recognised by Lord Eldon. In that case he was able to give costs, not *simpliciter* by virtue of the jurisdiction in lunacy, but because the property of the lunatic consisted in part of a fund in Chancery over which he had jurisdiction. He thought he might deal with that fund for the purpose of giving costs; but he recognised the doctrine of Lord Loughborough in the previous case. The reason of the doctrine is, I apprehend, that the jurisdiction is one of administering the property of the lunatic, and if this is once gone, then nothing remains in the hands of those who exercise the jurisdiction to administer. That being the doctrine previous to the statute 6 Geo. IV., c. 53, then the question is, how it is altered by that statute? The statute is entitled "An Act for limiting the time within which inquisitions of lunacy, idiotcy, and non compos mentis, may be traversed, and for making other regulations in the proceedings pending a traverse." Now, if the enactments of the statute are more extensive than indicated by this title, their effect would not be limited by the title, and the fact that the act is entitled only pending a traverse, would not be material. Let us see then, whether the enactments give any jurisdiction over the property of the lunatic, or the costs at any other time, except pending the traverse? If not, when once there is a verdict against the lunacy, the law would stand just as it did before the statute. Now, the 4th, which is the material section of the act, enacts, "that it shall be lawful for the Lord Chancellor, Lord Keeper, or Lords Commissioners, or other the person or persons entrusted as aforesaid, from time to time after the return of any such inquisition as aforesaid, and notwithstanding any petition or order which may be depending relating to a traverse of such inquisition, to make such orders relative to the custody and commitment of the person or persons, and commitment, management and application of the estates and effects of any person or persons who shall or may have been found lunatic, idiot, or of unsound mind by any such inquisition or inquisitions, as he or they shall think necessary or proper." Does that authorize the Lord Chancellor, or the persons acting in the jurisdiction of lunacy to deal with the property of the alleged lunatic after the trial of a traverse finding him not a lunatic? We have come to the conclusion (it being necessary to decide this, because if we had jurisdiction and could act, then we should be inclined to give costs) that we have no such authority given to us by the statute. There are two main grounds on which, after narrowly considering the words of the statute, we think that must be taken to be the result. We reject the words of the title to the statute, but we find exactly the same meaning in the words of the 4th section. The words are—"it shall be lawful for the Lord Chancellor," and so on, "from time to time after the return of any such inquisition, and notwithstanding any petition or order which may be depending relating to a traverse of such inquisition to make such orders relative" and so on, that is, notwithstanding any petition or other proceeding the Court may proceed. That is, you need not wait, as before the statute you must have waited, till the absolute establishment of the lunacy on the trial of the traverse, but you may, in the mean time, deal with the property on the foundation of the inquisition pending the further inquiry as to the lunacy. These considerations go far to show that the power given by the statute beyond what existed before was meant to be conferred only pending the trial of the matter upon the traverse. But that conclusion is made abundantly clear, when we see what a contrary construction must necessarily lead to. It must lead to this, that this jurisdiction is given to deal with the person as well as the property of the alleged lunatic. The enactment enables the court "to make such orders relative to the custody and commitment of the person and persons, and the commitment, management, and application of the estate and effects" of the alleged lunatic. These words are all of the same sentence, and are all governed by the words "it shall be lawful to make such orders." To what period then is it that that jurisdiction is meant to extend? Is it to the period up to the time when the non-lunacy is completely established, or is it to continue afterwards? If afterwards, it must be meant that the court should have power to deal with the person as well as the property; and on the other hand, if the court is not enabled to deal with the person, neither is it enabled to deal with the property.

The whole is one enactment, and the whole relates to one period. This, if agreed to, in effect is a *reductio ad absurdum*. We have come to the conclusion, therefore, that, not being authorized to act *cum imperio*, as Lord Loughborough expresses it in the case I have referred to, we can only deal with the case under the jurisdiction conferred by the statute, and that this statute does not authorize us to give any costs whatever. Then arises the other point which has been suggested, namely, that inasmuch as the party who was intended to be the committee and those who sued out the commission have property and papers in their possession belonging to the alleged lunatic, and the petition asks that they may be ordered to deliver up these, whether, although we could not make an order giving the costs, we could not make the delivery up of the property and documents conditional on payment of the costs. We have considered that, and we are of opinion, that we are not entitled to impose any such terms. The delivery up of the property and documents by the respondents, now that the alleged lunacy has been successfully traversed, is a matter of simple justice. The respondents took into their possession property and papers belonging to the alleged lunatic, because that was deemed necessary for his protection, by anticipation, as it were, upon the assumption that the result of the inquisition would be to establish a title to them in the respondents. In this state of things it was reasonable that they should hold them; but when, upon the result of the traverse, he is found not to be a lunatic, of course they are his own property, and he is entitled to the possession of them. Indeed, any attempt to impose such terms would be nugatory, and would leave the parties open to an action of trover, or other action, at the instance of the petitioner. The order, therefore, we make is an order to supersede the commission, and that the respondents deliver up the papers and property of the petitioner in their possession.

---

(Before the Lord Chancellor, January 19th and 26th, 1852.)

### Percival *v.* Caney.

*Answer of Lunatic's Committee.—Evidence of Lunatic's Administratrix.*

A bill was filed against a lunatic, and her three committees praying relief in respect to a particular fund. The lunatic put in her answer by her committees. The lunatic then died, and one of her three committees, and the wives of the other two took out administration to her estate. The suit being revived against the five, as representatives, they put in an answer referring to the former answer of the committees, and stated that to be their defence. The Court held that the admissions in the answer of the three, as committees, could be read against the five as representatives. The wife of one of the committees was examined in the original cause, as a witness on behalf of the whole of the defendants thereto, and the Court decided, that the husband being a defendant, as committee, had no such interest in the lunatic's estate as would prevent his wife being examined on behalf of the lunatic, and that therefore her evidence was admissible.

A bill was filed by Mrs. Percival, in 1847, against Miss Caney, a lunatic, and against Mr. Stanton, Mr. Verrell, and Mr. Page, the committees of her estate, praying that it might be declared that a sum of £4300 3 per cent. consols, in Miss Caney's hands, was held in trust for the plaintiff, and that the same might be transferred to her. Miss Caney, by her committees, answered the bill, and also the amendments, in which certain facts were admitted. Mrs. Page, the wife of one of the committees, was examined in that cause as a witness for the defendants. In 1849, Miss Caney died, and administration to her estate was granted to Mr. Stanton, one of the committees, and to Mrs. Verrell and Mrs. Page, the wives of the other two committees. A supplemental bill, and bill of revivor, was then filed against Mr. Stanton as an administrator, Mr. Verrell and his wife, and Mr. Page and his wife, the wives as administratrixes of the estate of Miss Caney. All these five then put in their answer, referring shortly to the former answer of the three, and craving leave to refer to the statements therein contained, and might be taken to have been restated in their present answer. At the rehearing of the cause upon appeal,

Mr. Russell and Mr. W. Wellington Cooper, counsel for the plaintiff, proposed to read the admissions in the answer of the three committees, Mr. Stanton, Mr. Verrell, and Mr. Page, to the original bill, against these three persons, and Mrs. Verrell and Mrs. Page, as the administrator and administratrixes of Miss Caney's estate.

Mr. Malins, Mr. Chandless and Mr. Steere, for the defendants, objected to the admissions in the answer being read, on the ground that the answers of the committees could not have been read against the lunatic herself, and could not now be read against the representatives of her estate in the supplemental suit.

Mr. Russell having replied,

The Lord Chancellor (Lord Truro) said: I am of opinion that it is competent for the plaintiff to read the answer to the original bill. The question does not necessarily arise whether the answers could or could not have been read against the lunatic, in the original cause. The arguments of the plaintiff were mostly directed to the point, that whatever might have been the effect of the answer as against the lunatic in the original cause, the circumstances of the case had been changed by the answer in the revived suit. It is said that the defendants to the revived suit had virtually set out in their answer to that the whole of the answer of the three defendants (the committees) in their answer in their original suit; and that answer is found at length on the same record. I must therefore, look to ascertain who the defendants to the revived suit are, and I find that the answer in the first suit was by the three committees of the lunatic, and the defendants in the revived suit are the administrator and the two administratrixes of the lunatic, and the husbands of the two administratrixes, to the number of five persons. That answer is the answer of the five, and they there say the plaintiff is not entitled to the decree she asks, for the reasons set forth in the answer of three of us to the original bill; and they refer to the answer of the three to the former bill, and crave to have the same benefit of the defence therein made, as if the same had been fully re-stated in the answer of the five to the supplemental bill, and that it may be taken as if they have pleaded the same matter therein. The record, therefore, contains the answer of the five defendants in the revived suit, and I consider the effect of that answer to be pretty much the same, in effect, as if they had said that their defence was contained in a certain deed or written document, and that document had afterwards been properly proved in the cause. If the defendants in the revived cause have so referred in their answer to all the matters set forth in the answer of the committees to the original bill, as to make such matters a part of their answer in the revived cause, then the plaintiff is entitled to read an admission from the first answer against the five defendants; and I am of opinion that they have done so, and that the plaintiff is entitled to read the committees' answer. It is not a question, whether the plaintiff can read the answers of the committees of the lunatic against the lunatic, but whether, where the defendants to a bill of revivor say their defence is contained in an answer of some of them to the original suit, and pray the benefit of the statements there made as if they had repeated them, the plaintiff has a right to read the first answer against them.

The admissions were then read, and the cause proceeded.

JANUARY 26TH.

Mr. MALINS, Mr. CHANDLESS, and Mr. STEERE, offered to read, as part of the defendant's evidence, the depositions of Mrs. Mary Anne Page, the wife of Mr. Page, he being one of the committees, and she one of the administratrixes, of the lunatic's estate. These depositions were taken in the original suit on behalf of the three committees, and on the hearing in the court below, the Vice-Chancellor (now Lord Justice) Knight Bruce had refused to receive the evidence.

Mr. RUSSELL and Mr. W. WELLINGTON COOPER contended that the evidence of Mrs. Page was inadmissible, she being the wife of a defendant. Mr. Page, himself, could not have been examined, having a material interest, inasmuch as he was liable to the costs.

Mr. MALINS, in reply, argued that the evidence was admissible, as that of a defendant under the provisions of Lord Denman's Act, 6 and 7 Vict. c. 85 : and that she was so also under the new Law of Evidence Act, 14 and 15 Vict. c. 99.

The LORD CHANCELLOR.—On the best consideration that I can give to this case, I think the evidence of Mrs. Page is admissible. The matter has been examined with great care in the argument, and it deserved to be so, and gives rise to questions of a peculiar kind. The first point is, whether Page, the husband, one of the committees, could have been examined. Now, it is one thing, whether a party may be a witness, and another thing whether he has pursued the proper course for enabling his evidence to be taken. In the case of Burton *v.* Langham, 5 C. B. Rep., I examined all the cases in reference to committees of lunatics, and came to the conclusion that a committee takes no estate or interest in the lunatic's property under the grant from the crown. I think, therefore, that in the present case the three committees had no interest in the estate. The bill seeks to charge the lunatic's estate, and contains nothing to charge the committee personally; and Mr. Page, therefore, stands as a defendant without interest, not even as a trustee, for he is not so much as a trustee, his position only amounting to that of a joint manager of the estate. But it is said that as one of the defendants to that suit he is liable for costs. Supposing that Mr. Page had any liability with regard to the costs, or, proceeding a step beyond that, that he is liable to the subject-matter of the suit, nothing can be clearer than the provision of the Act of Parliament that a defendant may be examined on behalf of a co-defendant, or on the behalf of the plaintiff, notwithstanding his interest in the matter in dispute. Whether, therefore, he is liable in respect of the subject-matter or for the costs, will form no objection to his being examined. It is said that he could only be examined under an order for that purpose, which would be an order made before the enactment which enabled a defendant to be examined. The question is, were the defendants entitled to such order? I think that Mr. Page had no interest in the original suit beyond that of his character of committee, and that he was entitled to such an order. But assuming that Mr. Page can be examined, does it follow that his wife can be examined? The long established rule of law is, that a wife cannot be examined for or against her husband; and no alteration has yet been made in that established rule of law. It is a rule founded on a principle which is more valuable even than the administration of justice—the necessity of preserving the confidence and happiness of domestic life. The liability to error through misconception, the preservation of the confidence which should exist between man and wife, supported the rule that a wife should not be called on to give evidence against her husband. Opinions have doubtless been expressed against that rule, but until the Legislature clearly abrogates* it, I shall not feel justified in departing

* This alludes to the statute 14 and 15 Victoria, chap. 99, entitled "An Act to amend the Law of Evidence."

from it. Therefore, though I am clearly of opinion that Mr. Page can be examined, I doubt whether, as a general rule, Mrs. Page can be examined also. But inasmuch as Mr. Page had no interest in the original suit, it seems to me that in no sense can the examination of Mrs. Page be said to be evidence against her husband, so as to bring it within the rule of law which excludes her testimony. I think, therefore, that she must be admitted as a witness. Looking at the prayer of the original bill, and the issue raised at the time Mrs. Page was examined, I am of opinion that she was not examined either for or against her husband. The circumstances which have subsequently occurred to change the character of the defendants in the revived suit, cannot affect the admissibility of that evidence. It seems to me, therefore, on a consideration of all the circumstances of the case, that the deposition of Mrs. Page can now be admitted and read as evidence.

---

(Before the Lords Justices of Appeal, Jan. 28th, 1852.)

Anderson *v.* Manson, and *In the matter of* Mr. James Manson.

*Answer and defence of a lunatic defendant.*

Where a lunatic who is so found, and who has had a committee of his estate appointed, is made a defendant to a suit in Chancery, he answers the bill and defends the suit, as a matter of course, by his committee, and no guardian is necessary. A gentleman, found a lunatic, was made a defendant as one of the next of kin of a deceased relation who had died intestate. The suit was for the administration of the estate. The lunatic and his committee presented a petition praying that the committee might be appointed guardian to defend the suit, and that a part of the estate might be sold to pay the costs to be incurred in the suit for the purpose of establishing the title of the lunatic as one of the next of kin; but the Court refused to make any prospective order as to the costs, and refused to appoint a guardian, but gave leave to the committee to defend the suit for the lunatic.

This was a petition presented by the lunatic, and the committee of his person and estate, Mr. W. Thacker. It stated that a commission had issued against Mr. James Manson, under which he had been found a lunatic, and that there had been the appointment of the committee; that the committee's accounts has been passed; that on the 20th of Dec., 1851, William Anderson and another filed a bill against the lunatic and other persons as next of kin of Mrs. Margaret Manson, deceased, who left personal estate divisible among her next of kin, praying that administration accounts might be taken, and the rights of the parties interested in her estate might be declared. The undisposed of residue of her estate consisted, it was said, of 6000*l.* The petition, as the petition of the lunatic, prayed that his committee might be appointed his guardian, by whom he might answer the bill and appear in the suit and prosecute his claim to be one of the next of kin of Mrs. Manson; and the committee prayed that he might be at liberty, as such committee, to take and concur with the other defendants in taking all necessary steps to prosecute the lunatic's claim as one of such next of kin, and that all such costs as should be properly incurred by both the petitioners in appearing to and answering the bill, and generally in the suit or otherwise relating or incidental to the lunatic's claim as next of kin, and as should not be paid out of the estate of Mrs. Manson, including the costs of the petition and consequent thereon, might be raised and paid out of the estate of the lunatic.

Mr. W. Collins appeared in support of the petition, which was unopposed.

He stated that the reason why the order was asked respecting the costs was, that the fund was supposed to be divisible into six parts, of which the lunatic was entitled to one; but as there had been a great number of persons belonging to the family of Mrs. Manson, it might be necessary to incur heavy costs in prosecuting extensive inquiries.

Lord Justice Lord Cranworth.—As to the anticipated extra costs of prosecuting these inquiries, the Court will of course give no directions. To ask for them now is crying out before you are hurt. If we give leave to defend the suit, which I am disposed to do, the committee will do so in the usual way; and if it shall appear that costs are incurred which the committee thinks should be raised out of the lunatic's estate, he may come here and say so, and we can deal with the matter. I am of opinion, that we ought not to make any prospective order as to costs.

Lord Justice Knight Bruce.—This petition is entitled as well in the cause as in the matter of the lunacy. That is, in my opinion, incorrect. That part of the petition which prays the appointment of a guardian is unnecessary. The lunatic having been made a defendant, will answer the bill, and defend the suit by his committee in the ordinary way, and quite as a matter of course. You may take the common order for leave to defend and reserve liberty to apply. The petition must be amended, by striking out all in the title but the lunacy.

---

(Before the Lords Justices of Appeal, January 14th and 21st, 1852.)

*In the matter of* Mr. Pattinson.

*Jurisdiction of the Lords Justices.*

It is doubtful whether the Lords Justices have jurisdiction to make an order, under the statute 13 & 14 of the Queen, c. 60, vesting the estate which has descended on the heir of a trustee, and which heir was a person of unsound mind, in a new trustee, the words of that act confining the authority "to the Lord Chancellor, entrusted by virtue of the Queen's sign manual."

Mr. J. V. Prior appeared in support of a petition, presented under the statute 13 & 14 of the Queen, c. 60 (the Trustee Act, 1850), commonly called Mr. Headlam's Act. The object was that an order should be made whereby the estate which had descended on a person of unsound mind, should be vested in another person as a trustee for the owner. This person was one not found a lunatic by inquisition, but was of unsound mind. Such a case was provided for by the statute in question, and their lordships being entrusted with the exercise of the jurisdiction in lunacy, were competent to make the order.

Lord Justice Lord Cranworth conferred for a short time with the Lord Justice Knight Bruce, and said, "Without meaning to say that we have no authority to make this order, there is a point well worthy of consideration before the order is made. The statute which constitutes this court of appeal, namely the 14 & 15 of the Queen, c. 83, enacts in the 13th section, that nothing shall affect the powers, duties, or authorities of the Lord Chancellor, by virtue of any appointment under the sign manual of the Crown, as having the custody of the persons and estates of lunatics. We are, it is true, also exercising a jurisdiction in lunacy by warrant under the Queen's sign manual, but the Act under which this petition is presented (like the preceding statute, 11 Geo. IV. and 1 Wm. IV. c. 60) only authorizes the Lord Chancellor, authorized by the Queen's sign manual, to make the order now asked. Every section says, "the Lord Chancellor authorized as aforesaid," so that it occurs to us a question whether we have authority; and that it would be a safer course to apply to the Lord Chancellor, who undoubtedly has authority to act. The statute does not, after the words Lord Chancellor, go on to say, " or other person or persons authorized as aforesaid." I repeat, we do not say we have not the power, but we consider the point one well worthy the petitioner's consideration.

Lord Justice Knight Bruce.—If this be the true construction, and I do not dissent from the view of my learned brother, all that we can do will do the petitioner no good. If the order is made by us, and the owner should ever have occasion to sell, he will have all the conveyancers to contend against: the title will be objected to. It rests with the Crown to say who shall be the person or persons to exercise these functions in lunacy. A warrant is directed in such way, as that the Lord Chancellor and ourselves, the Lord Chancellor alone or either of us with his Lordship, can adjudicate on such a matter, but then the statute called "The Trustee Act, 1850," speaks of the Lord Chancellor only. We have called your attention to the point, but if you like to hazard falling into the hands of the conveyancers, we may be disposed to make the order.

Mr. J. V. Prior declined, under the circumstances, to take the order; but said he would apply to the Lord Chancellor.

N.B. The case was brought before the attention of the Lord Chancellor (Lord Truro) on a subsequent day, when the order was made; his lordship observing, that without deciding whether the Lords Justices had or had not this jurisdiction, it was clear that he did possess it, and therefore he considered it the safer course that he should make the order. The inclination, however, of his opinion was, that the statute constituting the court of appeal, followed by the warrant under the sign manual, conferred on the Lords Justices every authority in lunacy which his lordship himself possessed.

---

(Before the Lords Justices of Appeal, January 26th, 1852.)

*In the matter of* Mr. Townsend.

*Arrangements for the Funeral of a deceased Lunatic.*

When a lunatic had died, and there was no committee of the person or of the estate, the Court directed the parties with whom the lunatic had resided, to proceed with the funeral, but declined to make any order as to payment of the expenses; directing a petition for such payment to stand over. The Court held, that for such a purpose a petition was necessary.

A petition was presented in this matter by the nephew, one of the next of kin, and the heir-at-law of Mr. Townsend, the lunatic, stating that the lunatic died three days since; that there was neither a committee of the person nor of the estate, although a person had been approved; that the petitioner and his wife, with whom the lunatic resided, had no money to pay for the funeral; and that the petitioner's wife who had had an allowance of 50*l.* a year out of the lunatic's estate, had not been paid any part since the death of the committee in October last. It was therefore prayed that a sufficient sum should be paid out of the money in Court to the credit of the lunacy, to defray the funeral expenses.

Mr. Grove supported the petition.

Mr. Follett, for other next of kin, appeared to oppose the petition as being entirely unnecessary. He also appeared for the person who had been approved as committee. It was only necessary in such a case to take out a warrant in the Lunatic Office, and the matter would have been arranged.

Lord Justice Lord Cranworth said, it appeared from the opinion of the officer who attended from the office, that a petition was necessary; and wished to know whether the ordinary preliminary step had been taken for the interment.

Mr. Grove said, that the nephew and his wife had done so, but had no money to pay the expenses; but were willing to complete the funeral. A will of the lunatic was sealed up and deposited in the Lunatic Office, and it was desired that it might be opened to ascertain whether it contained any directions.

Their Lordships directed the will to be opened, and that the funeral should take place; and that the petition should stand over to a future day. They declined, at present, to make any order for the payment of money out of court.

---

(Before the Lords Justices of Appeal. January 28, 1852.)

*In the matter of* Noble.

*A Lunatic's allowance permitted to be paid to a survivor of two committees, the Estate being very small.*

Two committees of a lunatic's estate were appointed, and one of his person. One of the two died, and the Court permitted the survivor to receive the dividends on the production of an affidavit of his solvency.

This was a petition praying that the dividends, amounting to about £100 a year, arising from consols, the only property of the lunatic, might be paid to the surviving of two committees of the estate to be applied by the committee of the person for the benefit of the lunatic. The petition stated that two committees of the estate had been appointed, one of whom had died, and that a committee of the person had been appointed.

Mr. Osborne, in support of the petition, urged, that as the estate was so small, it would be a great benefit if the expense of the appointment of a new committee were saved.

Lord Justice Lord Cranworth: Is there any affidavit as to the solvency of the surviving committee? It may be all very right to appoint two persons as committees and to entrust them with the lunatic's estate, but it may be far from proper that only one of them may be trusted. If such an affidavit is produced to the officer, I think the order may be made, to save expense, as the estate is so small.

Lord Justice Knight Bruce concurred, and directed the order to be made on the production of the affidavit.

Mr. Cottrell, on behalf of the next of kin, offered no opposition.

---

(Before the Lords Justices of Appeal. February 11th, 1852.)

*In the matter of* Mrs. Hewson.

*Allowances made to indigent members of a Lunatic's family out of her Estate.*

Mr. Stuart appeared in support of a petition in this matter, which stated that a petition was presented to the Lord Chancellor, Lord Cottenham, praying certain allowances to a nephew and niece, and a sister of the lunatic out of her estate. The Master in Lunacy had by his report approved of an allowance of £50 a year each to the sister and a niece of the lunatic, who were persons in very humble circumstances of life, and £200 a year to the nephew, who was a married man, with four children, and who had been a Church of England Missionary in India, and now desired to be ordained in that Church. On the hearing of the petition, Lord Cottenham would not approve of the allowances until he was informed whether, during her sanity, Mrs. Hewson had shown feelings of kindness and intentions of bounty towards her nephew, but the other allowances were confirmed. The point was now brought before the Court, but the affidavits failed to show that the nephew had been an object of Mrs. Hewson's bounty, but they also showed that the niece had never received benefits from her. The affidavits as to character, and as to the necessity of the nephew for such aid in his professional endeavours were satisfactory.

Mr. Shapter, for the next of kin, also supported the prayer of the petition.

Lord Justice Knight Bruce: Under all the circumstances of the case, we are of opinion that the allowance seems reasonable, and may be made. It appeared at first sight startling, that £50 a year should be considered sufficient for a sister of the lunatic, and so large a sum as £200 a-year be asked for her nephew.

